# Macrophage subtypes and pathways in autoimmune interstitial lung diseases: potential therapeutic targets

**DOI:** 10.3389/fimmu.2025.1638345

**Published:** 2025-10-01

**Authors:** Richa Tyagi, Surya Kant

**Affiliations:** ^1^ Department of Pulmonary Medicine, Sanjay Gandhi Post Graduate Institute of Medical Sciences (SGPGIMS), Lucknow, India; ^2^ Department of Respiratory Medicine, King George’s Medical University, Lucknow, India

**Keywords:** connective tissue disease, interstitial lung disease, macrophage polarisation, monocyte derived alveolar macrophage, antifibrotic agents, profibrotic macrophages, single cell RNA and transcriptome sequencing, macrophage genetic characteristics

## Introduction

Macrophages constitute a heterogenous population of innate immunity cells that exhibit dynamic plasticity and maintain tissue homeostasis. A dichotomous classification of macrophages exists, i.e., the classically activated proinflammatory M1 subtype and alternatively activated reparative M2 cells that are modulated by the tissue microenvironment, however, it is being realised that a continuum of phenotypes exists based on different stimulation factors, receptor profile and cytokine expression and a certain subtype dominates at a certain stage in the disease process based on signal from the surrounding tissue (1, 2). These phenotypes play an important role in pathogenesis of autoimmune diseases that are characterised by dysregulated innate immunity, activation of T-lymphocytes, autoantibody formation and development of interstitial lung disease due to ongoing abnormal inflammation as well as uncontrolled stimulation of fibrotic pathways. Targeted therapy directed at these pathways can be a supplicating strategy to avoid and limit pulmonary fibrosis in autoimmune disorders (3, 4).

## Macrophage origin, function and homeostasis

Macrophages are either yolk sac/fetal liver in origin or derived from bone marrow monocyte lineage (4). Most tissue-resident are embryonic in origin while brain microglia, dermal, intestinal macrophages and those recruited in inflammation arise from blood monocytes. Pulmonary alveolar and interstitial macrophages originate from yolk sac, though the latter can also be recruited (5–12).

Human alveolar macrophages are identified by the presence of sialoadhesin CD169, scavenger MARCO and interstitial macrophages by CD36, CX3CR1; both share the antigens HLA-DR, CD11b, CD11c, CD 14^low^, CD16^+^ and mannose receptor CD206. In resting conditions, the renewal of cells is dependent on CSFR-1, MCSF and IL-34 (13).

Macrophages exhibit TLRs, C-type lectin, dectin and NOD-like receptors on their surface (14). Alveolar macrophages are chiefly concerned with phagocytosis while interstitial macrophages are primarily involved in tissue homeostasis and immune regulation. Endogenous or exogenous signals such as apoptotic cells or microorganisms/irritants lead to recruitment of bone marrow derived monocytes to the lungs (13). Upon recognition of such triggers by surface receptors, macrophages engulf the particles incorporating it into phagolysome under influence of PI3K/Akt and mTOR pathways respectively, leading to its digestion (15).

**Figure 1:**
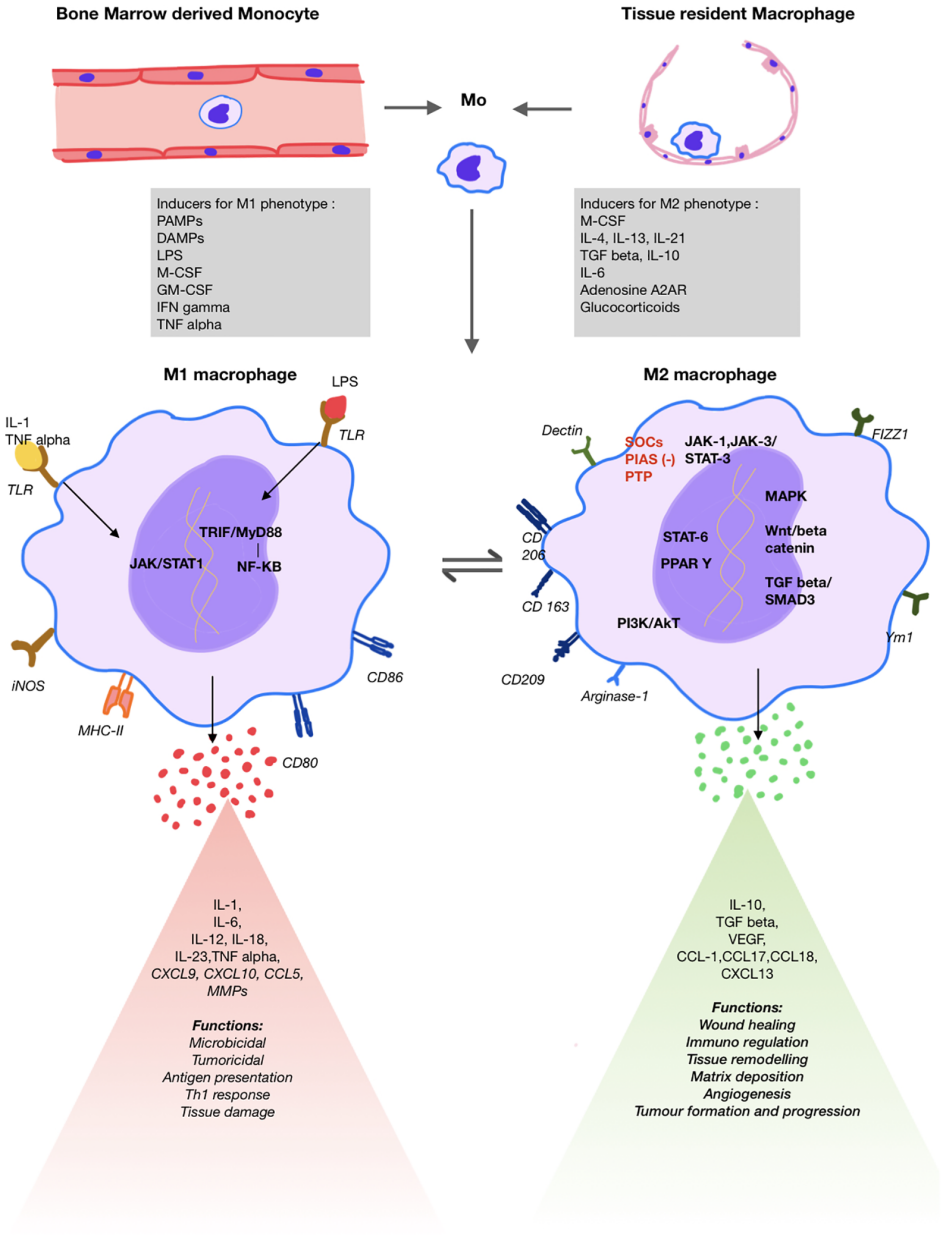
Schematic diagram showing macrophage origin, differentiation, key surface markers, signalling pathways, products released and respective functions of M1 and M2 macrophages.

Similarly, the dysfunctional cytoplasmic organelles are cleared through autophagy. Efferocytosis is accomplished through scavenger receptors and MERTK (13–16). This effective clearance of damaged and apoptotic cells is necessary to avoid exposure of autoantigens and thus development of autoimmunity. Concurrently there is release inflammatory cytokines (TNF-α, IL-1β, -6, -12, -18, -23) and chemokines (CXCL-1, CXCL-2) that recruit inflammatory cells and upregulate MHC-I expression on surrounding tissue cells thereby promoting autoantigens presentation to T-cells. They also exhibit CD86 and MHC II molecules to present antigens to T-lymphocytes thereby linking innate and acquired immunity. The macrophage produced degrade extracellular matrix releasing sequestered vascular endothelial growth factor promoting angiogenesis further amplifying inflammation (17, 18) (**Figure 1**).

With ongoing inflammation, there is activation of the profibrotic M2 phenotype to accomplish tissue healing and restore homeostasis (1, 2, 19). These cells produce anti-inflammatory cytokines such as IL-10 and TGF-β. The inflammatory cytokines IL-6 and IL-23 along with TGF-β promote T_H17_ differentiation of T-cells that play a crucial role in the pathogenesis autoimmune inflammation (20–22). Production of TGF-β is associated with increased fibroblast differentiation and fibrosis that plays an important role in development of ILD (23) (**Figure 1**).

Macrophages also carry out key metabolic roles. M1 macrophages convert arginine to nitric oxide via iNOS, promoting inflammation, while M2 macrophages turn arginine into proline and polyamines, aiding tissue repair (24). They also respond differently to hypoxia: M1 cells favor glycolysis, causing succinate accumulation, fatty acid synthesis, and inflammation through HIF-1α/IL-1β activation; M2 cells rely on oxidative phosphorylation, enhancing PDL-1 expression and T_REG_ differentiation while reducing IL-1β (24, 25). M2 macrophages also perform fatty acid oxidation, relevant in lipid-driven diseases like lupus, rheumatoid arthritis, and psoriasis.

Additionally, macrophages regulate iron metabolism by phagocytosing senescent RBCs. M1 cells retain iron promoting bacteriostasis, whereas M2 cells release it to support proliferation and matrix remodeling. High glutathione promotes M2 polarization, while low glutathione favors M1 activation for parasite defense (26–28).

## Pathways in macrophage activation and polarisation

Macrophage phenotypes have different gene expression, receptor and chemokine profile serving as markers for early identification. The M1 macrophages are activated by GM-CSF, Th-1 cytokines such as TNF-α, IFN-γ, IL-1, bacterial lipopolysaccharide, PAMPs and DAMPS. This triggers JAK/STAT1, nuclear factor kappa-β and IRF pathways resulting in expression of iNOS, MHC-II and SOCs-1 and proinflammatory cytokines such as IL-1,6,12, TNF-α and chemokines (3, 13, 18, 29) (**Figure 1**).

The anti-inflammatory M2 phenotype can be studied in four subgroups (2a, 2b, 2c, 2d) as studied *in vitro*. M2a cells play an important role in lung fibrosis. Triggered by Th2 cytokines (IL-4, IL-13) and M-CSF, they express innate scavenger receptor CD206, CD163, proteins like TGF-β, FIZZ1, arginase1 and chitinase-3 promoting fibroblast activation and CCL18 promoting collagen deposition. Insulin like growth factor-1 is also released that prevents myofibroblast apoptosis. M2b, stimulated by TLR and IL1R ligands, performs immunoregulatory function by producing high level of anti-inflammatory cytokine IL-10; along with proinflammatory cytokines such as IL-1β, IL-6, TNF-α, and low IL-12—making it the only M2 subtype to produce both anti- and pro-inflammatory cytokines. M2c, induced by TGF-β, IL-10 and glucocorticoids, is responsible for efferocytosis. M2d subtype is triggered by TLR, adenosine A2AR ligands and IL-6 and induces angiogenesis by promoting VEGF production (18, 30).

The M2 subtypes gene expression is controlled by the JAK1/JAK3 signalling pathways via STAT-3 activation which induces expression of anti-inflammatory genes. The STAT-3 pathway also cross talks with other key pathways including NF-kB, PI3K/Akt, Notch, Hedgehog, Wnt signalling and MAPK pathway. This pathway has negative regulators such as SOCs, PIAS and PTPs that are being explored for therapeutic use (30–32). Additionally, hydrogen peroxide generation by Cu/Zn superoxide dismutase leads to STAT-6 activation that triggers PPAR γ and δ transcription that promote fatty acid oxidation, mitochondrial biosynthesis and arginase-1 transcription respectively, hence, sustaining M2 phenotype (33, 34). PI3K/Akt, specific subtypes of interferon regulatory factors (IRFs) also modulate polarisation (35–37). Additionally, under hypoxia, HIF-1α promotes the production of profibrotic factors by upregulation of adenosine A2B receptor on M2 macrophages (3, 38).

Epigenetic regulation of polarisation also occurs as histone acetylation and methylation promote expression of fibrotic genes like IL1RA, MMP9, SPP1, CHI3L1, MARCK5 and PLA2G7 (39, 40).

There is increased recognition that a clear M1 and M2 differentiation does not exist *in vivo* rather there is a continuum of phenotypes. Single cell RNA sequence study of the healthy and fibrotic lung revealed several stages including monocytes (CD14+CD206^neg/lo^CD68^neg/lo^), intermittent transitional macrophages (CD14+ CD206^neg/lo^CD68^mid/hi^) and alveolar macrophages (CD14^neg/lo^ CD206^mid/hi^CD68^mid/hi^). PDGF-AA+ transitional and SPP^hi^ monocyte derived macrophages have been identified in fibrotic lungs. PDGF-AA promotes fibroblast proliferation and migration while osteopontin (SPP) promotes ECM deposition (41–43).

## Macrophages in autoimmune ILDs

Autoimmune disorders are characterized by formation of antibodies against self-antigens and dysregulated innate immunity. Although the exact mechanisms have not been elucidated in pathogenesis of autoimmune ILDs, macrophages being the chief cells of innate immunity, are involved in exaggerated inflammatory response, defective efferocytosis, presenting self antigens, and releasing profibrotic cytokines and chemokines.

Blood transcriptomic studies of fibrotic ILD patients have revealed overexpression of CD14^+^ monocytes that predict disease severity as well as mortality. The lineage of alveolar macrophages (MoAMs) is crucial as evidence shows their deletion markedly reduces the severity of bleomycin induced lung fibrosis in mice models while deletion of tissue resident macrophage has no such impact (44, 45).

**Figure 2:**
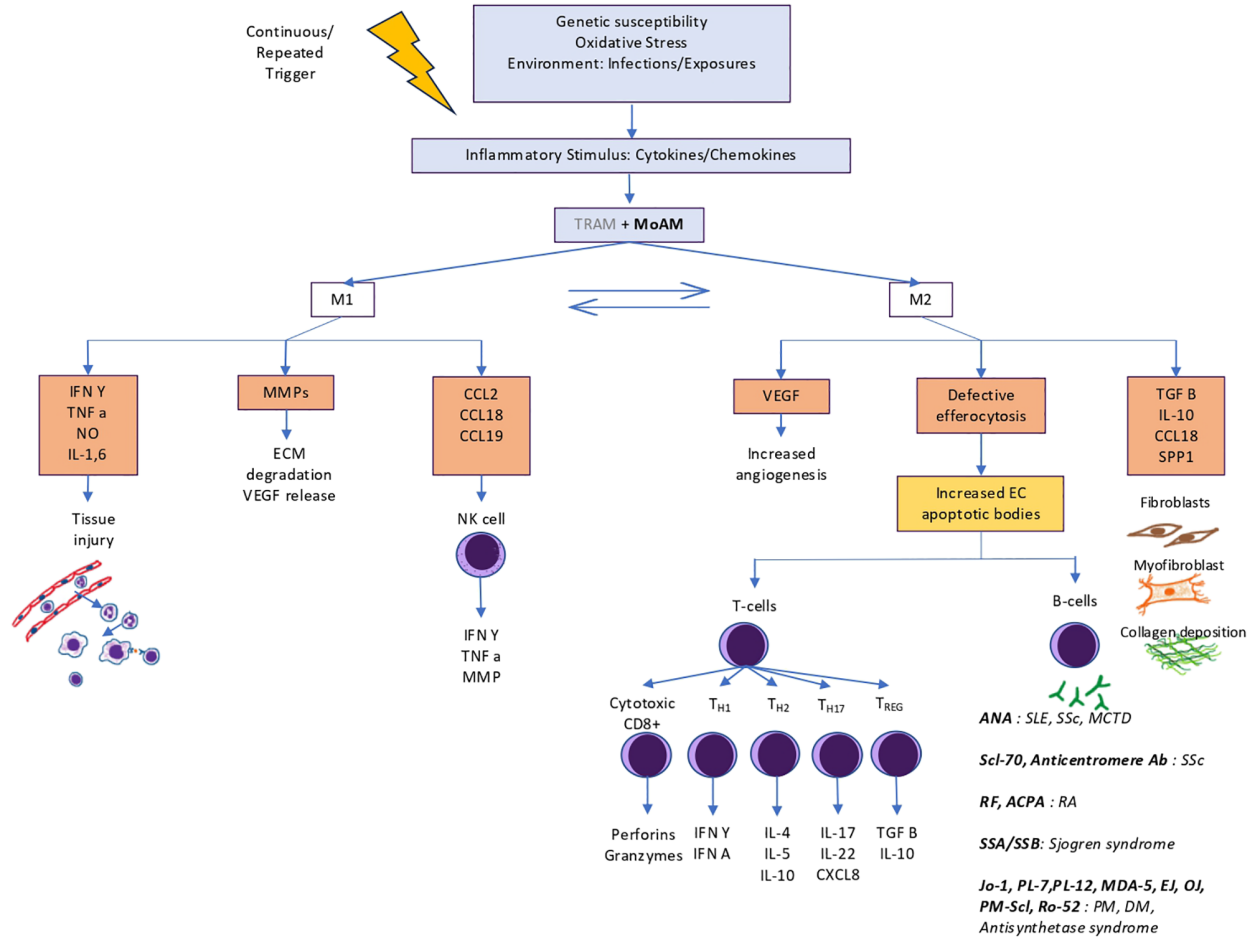
Role of macrophages in pathogenesis of autoimmune interstitial lung diseases. TRAMs play a limited role in initial inflammation and are mostly overtaken by MoAMs that convert into profibrotic macrophages. Failed efferocytosis leads to development of autoantibodies against intranuclear and antra cytoplasmic antigens that further perpetuates inflammation and dysregulated fibrosis.

General pathogenesis of autoimmune disorders is depicted in **Figure 2**. Key pathways of CTDs with high ILD prevalence are specified here.

The prevalence of ILD in rheumatoid arthritis patients is about 11% (46). In patients with RA-ILD, high peripheral monocyte count is associated with increased mortality (47, 48). The gene expression of these cells shows increased M1 polarisation in transcriptomics studies. There is upregulation of Dectin-1, IL-27, SOCs, IRF7 and JAK/STAT pathways. Epigenetically, glycotransferase guanylate binding protein 5 is overexpressed that promotes IFN-γ induced M1 typing (49–58). The enzyme peptidylarginine deiminases which mediates citrullination is regulated by PI3K/Akt signalling. Interestingly, this pathway is inhibited by syndecan-2 (SDC-2) an M2-associated CD148 ligand with antifibrotic effect. Also, in alveolar epithelium SDC-2 promotes caveolin mediated TGF-β receptor 1 degradation thereby preventing TGF-β mediated apoptosis (59–61).

ILD can be seen in roughly half the patients with systemic sclerosis (46). RNA sequencing in lung fibrosis mice models have demonstrated markedly increased M2 population (62). IL-4,13 and 10 promote M2 polarisation via STAT3 pathway activation. Additionally, CpG-binding domain 2 and MMP28 are significantly upregulated in SSc that promote PI3K/Akt signalling for M2 differentiation. Redox enzymes such as Sart-1 and Cu/Zn-SOD promote M2 phenotype by enhancing STAT-6 signalling (63–67).

Inflammatory myopathies have a high prevalence of ILDs ranging between 50% to 90% (68). This group is notorious for rapidly progressive ILDs and thus require urgent measures to combat inflammation and fibrosis (46, 69). Soluble CD206, a marker for M2 macrophages is highly elevated in patients of dermatomyositis ILD (69). Additionally, there is increased expression of IFN-γ and TNF- α suggestive of increased M1 polarisation early on in the disease (70–72). Ergo, there is possible involvement of different subtypes at different stages of the disease.

## Potential targets

The following aspects of macrophage physiology may be targeted to prevent pulmonary fibrosis in autoimmune disorders.

- Inflammatory cytokines: Methyl palmitate is a promising drug that inhibits macrophage activation, reduces TNF-α level and reduces fibrosis (73, 74). Anti-IL-6 Tocilizumab has been demonstrated to preserve lung function in patients with systemic sclerosis (75). IL-27 inhibitors are currently are undergoing trial for cancer immunotherapy and may be utilised for ILD (50).
- Recruitment: Nintedanib is an existing triple kinase inhibitor that has inhibitory action on CSF-1R as well and blocks M2 polarisation (76).
- Signalling pathways: Tacrolimus, Ruxolitinib inhibit the JAK/STAT pathways supressing the profibrotic M2 pathway (63, 65, 75). JAK inhibitor Tofacitinib has demonstrated positive results in IIM-ILD (77). Akt-1 pathway promotes ROS and TGF-β. Its deletion is associated with apoptosis of alveolar macrophages and prevention of lung fibrosis. Clevudine is a purine analog that can block Akt signalling (78). Syndecan is another potential agent inhibiting Akt pathway with demonstrated antifibrotic effect in human lung homogenates (59, 60).
- Fibroblast activation: Traditional Chinese medicine like Schisandra chinensis and Resveratrol have anti-TGF-β activity and hold excellent potential (79, 80). Pirfenidone has anti-TGF-β1 action and prevents M2 polarisation (81). PRI-724 reduces TGF-β gene expression by inhibiting B catenin signalling thus inhibition collagen deposition (82). Niclosamide, an antiparasitic drug shows promise in inhibiting Wnt/β catenin and TGF-β pathways (83). Another potential target for inhibition is S100A4, a M2 macrophage produced calcium binding protein involved in fibroblast proliferation (84). Fresolimumab, anti-TGF antibody has been beneficial in SSc (85). Microcystin-leucine arginine is another agent that prevents epithelial to mesenchymal transition (86). Imatinib loaded gold particles have been shown to inhibit macrophage and fibroblast activation (87).
- Macrophage apoptosis: BCL-2 inhibitors promote apoptosis and have demonstrated resolution of fibrosis in mice (88).

## Discussion

The first step in the development of lung fibrosis in most autoimmune diseases is damage of alveolar epithelium and inflammation followed by continuous TGF-β signalling resulting in epithelial to mesenchymal transformation (87). Macrophages are involved in pathogenesis right from excessive monocyte recruitment, MHC-I upregulation, T-lymphocytes activation, T_H17_ differentiation, impaired autophagy and apoptosis to activation of profibrotic pathways. These are highly plastic cells with potential to switch from one phenotype to another at different developmental stage of the disease process depending on the stimulus. Exaggerated response at any stage can contribute to chronic disease. The bivalent macrophage classification does not justify their heterogenous genetic and functional spectrum. With advent of scRNA sequencing transcriptomics, macrophages have been genetically characterised in fibrotic ILDs, their lineage traced to blood monocytes and prognostic markers identified. More importantly, the high risk genes have been identified from peripheral blood monocytes also. Similarly, genetic studies in autoimmune diseases can help identify the high risk biomarkers and better understanding of pathogenesis.

Several immunomodulatory and antifibrotic drugs are under study. Rather than upstream targeting like CSFR-1 that poses risks of widespread immune dysregulation, specific signalling pathways may be halted. The signalling pathways have complex interactions and different isoforms of molecules perform different actions thus inhibition of complimenting pathways simultaneously may yield better results in terms of fibrosis containment (63, 65, 75, 76). JAK/STAT kinase inhibitors not only prevent pulmonary fibrosis but also fibrosis in other organs expanding benefits (83). Anti-TGF-β agents as well as negative regulators of profibrogenic pathways are already under study (79–83). Targeting autophagy is a promising area for drug development to regulate the tissue microenvironment (89). Concomitant impact of metabolic and oxidative state needs to be studied in further detail to develop therapy directed at lipid mediators such as prostaglandins (24–28, 38).

Our knowledge has only recently grown in understanding the diverse landscape of macrophages. As MoAMs are the culprit cells responsible for promoting lung fibrosis, genetic/epigenetic modulation holds promise for precise targeting while sparing the homeostatic function of the resident macrophages. Novel agents targeting profibrotic macrophage are still in experimental stage. Application of scRNA sequencing to lung lavage/tissue/blood samples of different autoimmune disorders holds great promise for improving the understanding, prevention and better management of lung fibrosis.
